# Objectively measured preoperative physical activity is associated with time to functional recovery after hepato-pancreato-biliary cancer surgery: a pilot study

**DOI:** 10.1186/s13741-021-00202-7

**Published:** 2021-10-04

**Authors:** Caspar F. Mylius, Wim P. Krijnen, Tim Takken, Daan J. Lips, Hasan Eker, Cees P. van der Schans, Joost M. Klaase

**Affiliations:** 1 Research Group Healthy Ageing, Allied Health Care and Nursing, Hanze University of Applied Sciences, Petrus Driessenstraat 3, 9714 CA Groningen, The Netherlands; 2 Child Development and Exercise Center, University Medical Center Utrecht, Utrecht, The Netherlands; 3 Department of Surgery, Medical Spectrum Twente, Enschede, The Netherlands; 4 Department of Surgery, Medical Centre Leeuwarden, Leeuwarden, The Netherlands; 5 Department of Rehabilitation Medicine, University Medical Center Groningen, University of Groningen, Groningen, The Netherlands; 6 Health Psychology Research, University Medical Center Groningen, University of Groningen, Groningen, The Netherlands; 7 Department of Hepatobiliary Surgery and Liver Transplantation, University Medical Centre Groningen, Groningen, The Netherlands

**Keywords:** Hepato-pancreato-biliary cancer, Perioperative, Preoperative, Physical activity, Time to functional recovery

## Abstract

**Background:**

Surgical resection is currently the cornerstone of hepato-pancreato-biliary (HPB) cancer treatment. A low preoperative aerobic fitness level has been identified as a modifiable risk factor associated with complications after major abdominal surgery. A person’s aerobic fitness is influenced by performing moderate to vigorous physical activity (MVPA). This study aims to determine the activity monitor measured levels of MVPA performed among patients on the waiting list for HPB cancer surgery and their association with postoperative outcomes.

**Methods:**

A prospective, observational multi-center cohort pilot study was conducted. Patients enlisted for resection surgery on suspicion of HPB (pre)malignancy were enrolled. Performed MVPA was measured by an Actigraph wGT3X-BT. Additionally, aerobic fitness was measured via the Incremental Shuttle Walk Test, and (post)operative variables were collected from the electronic patient files. The association between MVPA and the pre- and postoperative variables was determined by univariate and multivariable (logistic) robust regression.

**Results:**

A total of 38 participants, median age 66.0 (IQR 58.25–74.75) years, were enrolled. The median daily MVPA was 10.7 (IQR 6.9–18.0) min; only 8 participants met the Dutch MVPA guidelines. Participant’s age and aerobic fitness were associated with MVPA by multivariable statistical analysis. Time to functional recovery was 8 (IQR 5–12) days and was associated with MVPA and type of surgery (major/minor) in multivariable analysis.

**Conclusion:**

Seventy-six percent of patients enlisted for resection of HPB (pre)malignancy performed insufficient MVPA. A higher level of MVPA was associated with a shorter time to functional recovery.

**Supplementary Information:**

The online version contains supplementary material available at 10.1186/s13741-021-00202-7.

## Introduction

Hepato-pancreato-biliary (HPB) cancer is a frequently diagnosed disease with an incidence of 248,800 patients diagnosed with HPB cancer in Europe in 2018, of which pancreatic cancer constituted the majority with 132,600 diagnoses (Ferlay et al. 2018). Since advancing age of the population is the most important factor contributing to the incidence of pancreatic cancer, the incidence and the average age of HPB cancer patients are set to increase in the coming years due to increasing life expectancy (Bray et al. 2018; Kontis et al. 2017). Surgical resection and adjuvant therapy are currently the cornerstone of treatment for HPB cancer (Kommalapati et al. 2018). Currently, approximately 20–30% of patients develop major postoperative complications which lead to increased length of hospital stay (LOS), decreased postoperative quality of life, and delay to chemotherapy (Pinto et al. 2016; Pearse et al. 2012; Kumar and Garcea 2018). Since complications and mortality rates following pancreatic and liver surgery increase with advancing age (Raill 2009), identifying modifiable risk factors in HPB cancer patients may help to reduce postoperative complications, LOS, and hospital costs (Straatman et al. 2015).

Preoperative aerobic fitness level has been identified as a modifiable risk factor in a variety of patients who need surgery (Snowden et al. 2010; Simões et al. 2018; Van Beijsterveld et al. 2019). A person’s aerobic fitness reflects the physiological reserve available to endure the physical stress of surgery and postoperative recovery (Levett and Grocott 2015). Low preoperative aerobic fitness is associated with negative postoperative outcomes such as prolonged LOS and increase in incidence of unplanned readmissions, morbidity, and mortality after major intraabdominal surgery (Moran et al. 2016a; Chandrabalan et al. 2013). A person’s aerobic fitness is influenced by his or her physical activity (PA) level (Hallal et al. 2012; Chastin et al. 2018). Consequently, current (inter)national guidelines for PA advocate to spend at least 150 min per week in activities with a moderate to vigorous intensity (MVPA) (Weggemans et al. 2018; WHO 2009).

Multiple studies investigated the relation between preoperative (self) reported PA levels and outcome after surgery concluding that a higher preoperative level of PA is not significantly associated with the presence of postoperative complications (OR=2.60; 95% CI=0.59 to 11.37). However, it has been previously reported that PA is significantly associated with shorter LOS following abdominal surgery (OR=3.66; 95% CI= 1.38 to 9.6) (Steffens et al. 2019). Nevertheless, correlations between self-reported PA and actual PA are generally low-to-moderate and ranging from *R*= −0.71 to 0.96 (Colley et al. 2019; Prince et al. 2008). Furthermore, previous studies have demonstrated that cancer patients overestimate their self-reported PA level when compared to objective measures (Vassbakk-Brovold et al. 2016).

Therefore, insight into the level of actual, objectively measured, PA and subsequent postoperative outcomes in patients scheduled for HPB cancer surgery is needed. This study aims to determine the activity monitor measured levels of MVPA performed among patients on the waiting list for HPB cancer surgery. Additionally, the secondary aim of the study is to determine the association between preoperative MVPA and the association with postoperative outcomes.

## Methods

### Study design and study population

This prospective, observational multi-center cohort pilot study was performed at the University Medical Centre Groningen (UMCG), the Medical Center Leeuwarden (MCL), and the Medical Spectrum Twente (MST) in the Netherlands. All centers are connected via a Managed Clinical Network HPB surgery. Ethical approval was obtained from the Central Ethics Review Committee of the UMCG under registration number 201800539, and all participants provided written informed consent. The primary objective of the study was the total of activity monitor measured MVPA performed by subjects in 1 week while awaiting HPB cancer surgery. The secondary objective was (1) the association between the subject characteristics and the performed MVPA, and (2) the associations between these parameters and the surgery outcome.

The research population consisted of adult (18 years and older) patients scheduled for resection of HPB (pre)malignancy between October 2018 and September 2019. Exclusion criteria were (1) receiving an intervention aimed at influencing PA in the preoperative period. Performing health-enhancing physical activity (e.g., fitness, jogging) on own initiative was allowed since this is part of the participants normal PA behavior; (2) receiving neo-adjuvant chemo(radio)therapy during the measurement period.

Potential participants who met the inclusion criteria were identified by the responsible surgeon directly after surgery enlistment and were invited to participate immediately after being informed about their pending surgical procedure. If eligible, potential participants received instructions on the purpose of the study and were provided an information letter. After giving informed consent, participants were visited at home to perform measurements and provide the activity monitor. After surgery, participants were treated by the Enhanced Recovery After Surgery protocol as part of the care as usual.

### Data collection

The primary outcome of the study was the total of activity monitor measured MVPA performed by subjects in 1 week while awaiting HPB cancer surgery. The secondary outcomes were (1) subject characteristics, (2) aerobic fitness, and (3) the functional recovery.

After informed consent, baseline characteristics were collected: age, height and weight, BMI (formula: weight/height^2^), smoking behavior (yes/no), occupation (work/volunteer, yes/no), and living (alone/together), education (lower/ higher), and alcohol consumption status. Alcohol consumption was coded as above the norm or equal to/below norm of a maximum of one consumption per day as defined by the Dutch health council (Health Council of the Netherlands 2015). Lower education was defined as (preparatory) vocational or primary education and higher education as (preparatory) academic or higher education.

Aerobic fitness was measured directly after providing informed consent. This was measured using the Incremental Shuttle Walk Test (ISWT) to determine the influence of aerobic fitness on the PA level. As an externally paced walk test, the ISWT yields greater physiological responses in comparison with self-paced walk tests (Singh et al. 1992). The test was performed once, in accordance with the Singh protocol (Singh et al. 1992). The maximum walking distance expressed in meters and the percentage of the predicted distance based on Probst et al. were used to determine a participant’s aerobic fitness level (Probst et al. 2012). Conventionally, the variability between healthy subjects is taken to be a standard deviation of 10%; the normal predicted range would be from 80 to 120%. Therefore, participants reaching a distance below 80% of the predicted distance were labeled unfit (Stanojevic et al. 2010).

MVPA level was measured using a hip-worn activity monitor; the Actigraph wGT3X- BT+ (Actigraph, Pensacola, FL, USA) was provided (Knaier et al. 2019; Sasaki et al. 2011; Moran et al. 2016b). The measuring period started the day after baseline characteristics were collected and lasted 7 consecutive days. Instructions for use included performing regular PA as they were used to and wearing the device during waking hours to minimize influencing sleep quality. The used cutoff counts per activity intensity level were sedentary time (<100 counts/min) and moderate (2020–5999 counts/min) and vigorous intensity PA (≥5999 counts/min) with 100 Hz measurement epoch (Troiano et al. 2008). The total amount of MVPA is determined both as the daily median of total accumulated minutes and as the daily median minutes accumulated in at least 10-min bouts [19], where the latter is generally defined as a 10-min period with an interruption of no more than 2 min below the threshold of 2020 counts per minute [28]. MVPA measured in 10-min bouts was used for further analyses. To identify non-wear time, the algorithm of Choi et al. was used (Choi et al. 2011). This algorithm defines non-wear times as periods of consecutive 0-counts for the duration of 90 min. A minimum of 6 measurement days or more had to be completed to be included in the analysis. Participants who wore the activity monitor for less than 6 days or did not undergo resection were excluded from analysis.

After completion of the activity tracker measurement week, the symptom burden of the past 24 h was determined by completing a translated version of the “MD Anderson Symptom Inventory” (MDASI) questionnaire. The MDASI median scores and the sub-domain “symptom burden” and “activity interference” scores were used to determine the participants’ symptom burden (Cleeland et al. 2000). Median scores are used per sub-domain.

After surgery, characteristic data of the surgery and outcome were collected from the electronic patient files. These included the surgery type (target organ, major/minor surgery, open/laparoscopic surgery). Major surgery was defined as any pancreatic or liver resection of at least three liver segments (Stanojevic et al. 2010). Mortality was defined as in-hospital all-cause mortality or within 30 days after discharge. Overall complications consisted of all surgical and non-surgical complications within 30 days of surgery. Major complication was defined as any Clavien–Dindo grade ≥ III complication (Dindo et al. 2004).

In the post-surgery phase, functional recovery was determined as the number of days between surgery and the day that adequate pain control requiring oral analgesia only was reached without signs of active (wound) infection, tolerance of solid foods, and independent mobility sufficient to perform activities of daily living at the preoperative level (Wong-Lun-Hing et al. 2017). LOS was determined at discharge and expressed in days between surgery and hospital discharge.

### Statistical analyses

Due to the pilot design of the study, the sample size target was 50 participants. This was based on comparable studies in major abdominal surgery including other organs aimed at determining preoperative PA behavior (Steffens et al. 2019). Statistical analyses were performed using the R software version 3.6.1 (R Development Core Team 2013). A *p*-value ≤ 0.05 was considered significant. Continuous data were summarized by median and interquartile range (IQR) and categorical data by frequency and percentage. Range was reported if deemed relevant.

MVPA data are frequently non-normally distributed due to outlying observations for a few persons having PA levels away from the bulk of the data. Therefore, a robust regression approach was undertaken throughout this study. Robust regression is a regression method suitable for non-normally distributed data with outliers; this method prevents a large influence on the association coefficients by outlying observations (Yohai et al. 1991; Koller and Stabel 2011; Hubert and Rousseeuw 1997). All enlisted patients, recording 6 or more measurement days, were used in the MVPA analyses; participants only receiving an exploratory laparotomy or laparoscopy without resection and those who were eventually not operated upon were excluded from the complication’s analyses. Furthermore, all participants that reached discharge from hospital were included in the time to FR analysis.

The association between the level of MVPA in 10-min bouts and the preoperative variables, and time to FR and pre- and perioperative variables was determined by univariate and multivariable robust regression (Portney and Watkins 2009). Furthermore, univariate and multivariable robust logistic regression was used to determine the odds ratio (OR) of the occurrence of complications based on the preoperative and per-operative variables (Koller and Stabel 2011; Hubert and Rousseeuw 1997; Portney and Watkins 2009). All multivariable analyses were performed using the measured independent explanatory variables identified to potentially have a significant association with the dependent variable from univariate regression analysis. Lastly, a subset analysis was performed to determine the association between MVPA in 10-min bouts and time to FR within the major complications group via univariate robust regression. LOS analysis is reported in the supplementary material.

## Results

**Fig. 1:**
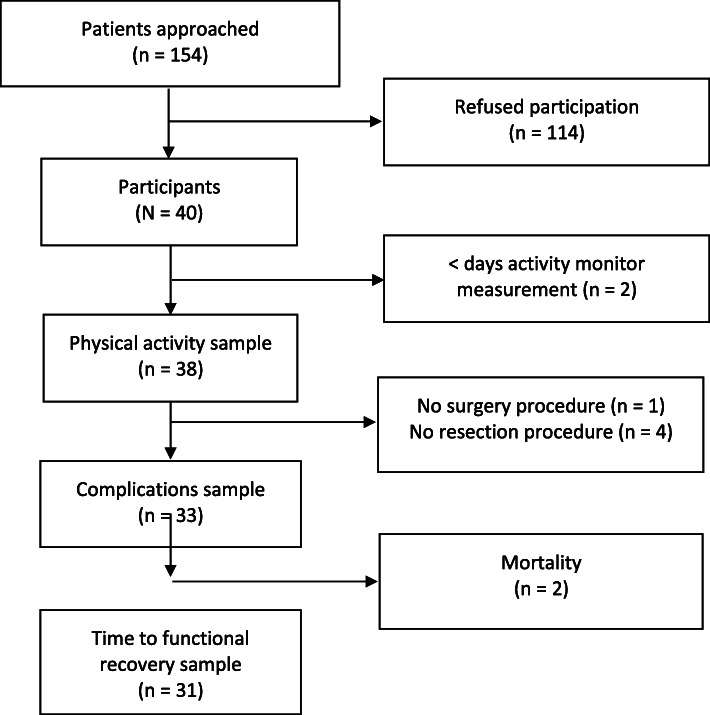
Inclusion flowchart

A total of 154 patients who met the inclusion criteria were approached for participation; 40 patients (26%) consented to participate in the study. Two participants were excluded from PA analysis due to not meeting wear-time criteria; the measurements from the remaining 38 participants were used for further analysis. Five participants had either no surgery procedure (one participant) or received a procedure without resection (exploratory laparotomy only, four participants). These patients were excluded from complication analyses. Furthermore, two participants were excluded from the time to FR analyses due to postoperative mortality. Figure 1 displays the flowchart of participant inclusion. Median time between placement on surgery awaiting list until baseline measurements and between baseline measurements, including the start of the activity monitor period, until surgery day was 0 days (IQR 0–0.75, range 0–14) and 31.5 days (IQR 22.25–45, range 9–171) respectively.

### Characteristics

Of the 38 participants, 22 participants were male, and the mean age of participants in both the PA and surgery outcome group was 65.8 years (±9.4) and 65.5 years (±9.8), respectively. The label unfit was given to 22 participants, with a median 69% (±31%) distance covered of the predicted ISWT distance in the PA group and 65% (±28%) in the surgery outcome group. Of the 33 participants that underwent the surgery procedure, 10 developed major complications. Participant characteristics and perioperative data are presented in Table 1.

**Table 1:** Patient characteristics

Variable	Physical activity sample (*N*=38) *N* (%) or median (IQR)	Surgery outcome sample (*N*=33) *N* (%) or median (IQR)
**Gender**		
Female	16 (42%)	14 (42%)
Male	22 (58%)	19 (58%)
**Age** (years)	66 (58.25–74.75)	66 (56–74)
**Height** (cm)	173 (167.8–182.8)	173 (167–182)
**Weight** (kg)	77 (70.1–87.6)	77 (70.5–88)
**BMI** (kg/cm^2^)	24.9 (22.8–28.2)	24.8 (22.7–28.3)
**Living situation**		
Living alone	8 (21%)	7 (21%)
Living together	30 (79%)	26 (79%)
**Educational level**		
Lower education	23 (61%)	19 (58%)
Higher education	15 (39%)	14 (42%)
**Work**		
Employed	14 (37%)	12 (36%)
Unemployed	24 (63%)	21 (64%)
**Alcohol consumption**		
Above norm	10 (26%)	9 (27%)
Equal or below norm	28 (74%)	24 (73%)
**Smoking**		
Yes	5 (13%)	5 (15%)
No	33 (87%)	28 (85%)
**MDASI total** (sum score)	1.87 (±1.75) (*N*=32)	1.86 (±1.85) (*N*=28)
Symptoms	1.81 (±1.68) (*N*=32)	1.84 (±1.81) (*N*=28)
Activity	1.91 (±2.29) (*N*=32)	1.92 (±2.30) (*N*=28)
**ISWT** (m)	430 (310–473.1)	430 (280–620)
Percentage of predicted (%)	69 (±31)	65 (±28)
Labeled fit/unfit	16/22 (42%/58%)	14/19 (42%/58%)
**Physical activity** (min)		
Time spend sedentary (per day)	564.45 (310.4–662.4)	580.4 (417.0–668.3)
Time spend sedentary (per week)	3951.1 (2172.8–4636.6)	4062.8 (2918.9–4678.5)
MVPA (total accumulated per day)	26.4 (16.8–43.8)	24.4 (16.6–34.5)
MVPA (total accumulated per week)	184.8 (117.7–306.9)	170.6 (116.1–241.2)
Adherence to guideline (yes/no)^a^	21/17 (55%/45%)	17/16 (48%/52%)
MVPA (total 10-min bouts per day)	10.7 (6.9–18.0)	11.6 (7.5–21.3)
MVPA (total 10-min bouts per week)	74.7 (48.3–125.94)	81 (52.6–148.9)
Adherence to guideline (yes/no)^b^	8/30 (21%/79%)	8/25 (24%/76%)
Wear time in percentage	66% (±27.6)	69.2% (±26.7)
**Highest Clavien–Dindo rating**		
No complication		16 (49%)
Minor/major		7/10 (21%/30%)
Grade I		2 (6%)
Grade II		5 (15%)
Grade IIIa		3 (9%)
Grade IIIb		5 (15%)
Grade IV		0 (0%)
Grade V		2 (6%)
**Target organ**		
Pancreas		13 (39%)
Liver		20 (61%)
**Type of procedure**		
Open		24 (72%)
Laparoscopic		9 (28%)
**Procedure size**		
Major		18 (54%)
Minor		15 (46%)
**Length of hospital stay** (days)		9 (7–15)
**Time to functional recovery** (days)		8 (5–12)
**Mortality**		2 (6%)

*BMI* Body mass index, *MDASI* MD Anderson Symptom Inventory, *ISWT* Incremental Shuttle Walk Test, *PA* Physical activity, *MVPA* Moderate to vigorous physical activity, *avg.* Average

^a^150 min per week accumulated total bouts

^b^150 min per week accumulated 10-min bouts

### Physical activity

The participants’ median level of MVPA was 10.7 min per day, wearing the activity monitor 66% (±29%) of waking hours per day. The MVPA variability between participants was large, ranging from 0 to 60.1 min per day. Eight participants (21%) met the PA guideline of 150 min MVPA per week. The level of MVPA reduced with 0.52 min per advancing age year, (*R*^2^= .31, *p*=.001), and increased by .02 min per m covered during the ISWT (*R*^2^ = .35, *p* = .008), and subjects labeled as fit (7.90 min more in fit subjects, *R*^2^= .20, *p*=.023) were identified as correlating with MVPA via univariate robust regression. Since the aerobic fitness level was derived from the ISWT distance covered, this variable was omitted from multivariable regression. The multivariable regression model for performed MVPA determined by multivariable robust regression was 29.05 + (ISWT ^(meters)^ * 0.01) + (age ^(years)^ * −0.35) (adj. *R*^2^= .41). The association between MVPA and preoperative variables via univariate and multivariable robust regression is displayed in Table 2.

**Table 2:** Uni- and multivariate robust regression association between preoperative variables and moderate to vigorous physical activity (10-min bouts)

Variable	Estimate	Std. error	***R***^**2**^	***t***-value	***p***-value	Adj. ***R***^**2**^
**Gender** (female)	−2.140	2.867	**0.016**	−0.746	.460	
**Age** (years)	−**0.521**	**0.146**	**0.309**	−**3.565**	.001*	
**BMI** (kg/cm^2^)	0.358	0.642	0.0332	0.557	.581	
**Living situation** (together)	−0.999	4.335	0.002	−0.230	.819	
**Work status** (employed)	5.491	3.686	0.092	1.490	.145	
**Education** (high)	5.045	3.153	0.083	1.600	.118	
**Smoking status** (no)	1.263	2.771	0.002	0.456	.651	
**Alcohol norm** (above)	5.935	3.415	0.101	1.738	.091	
**MDASI** total (avg, *n*=32)	−1.058	0.848	0.045	−1.247	.222	
**MDASI** symptoms (avg, *n*=32)	−0.884	0.823	0.031	−1.074	.291	
**MDASI** activities (avg, *n*=32)	−0.865	0.579	0.050	−1.496	.145	
**ISWT** (m)	**0.020**	**0.007**	**0.346**	**2.796**	.008*	
**ISWT** (% of predicted)	0.091	0.050	0.112	1.811	.078	
**Aerobic fitness** (labeled fit)	**7.905**	**3.325**	**0.204**	**2.378**	.023*	
**Multivariate**						
Constant	29.048	11.107		2.615	.013*	.414
**Age** (years)	−0.348	0.135		−2.576	.014*	
**ISWT** (m)	0.013	0.006		2.238	.031*	

*BMI* Body mass index, *ISWT* Incremental Shuttle Walk Test, *MDASI* MD Anderson Symptom Inventory, *avg.* Average

*p ≤.05

### Complications

Seventeen participants (51%) had complications of which ten (30%) were major. The association found between MVPA and the presence of major complications (OR = 0.99, 95% CI= 0.95–1.04, *p*= .703) was not statistically significant. A statistically significant association was found between the presence of major complications and BMI (OR = .71, 95% CI= 0.52–0.98, *p*= .036), % of predicted ISWT (OR= .98, 95% CI .97–.99, *p*=.008), and surgery type (OR = .24, 95% CI = 0.06–0.95, *p*= .043). The odds of major complications decrease with increasing BMI, more distance covered on the ISWT compared to the predicted distance, and a minor surgery procedure. The OR from multivariable robust logistic regression including surgery type and ISWT ^(% of predicted)^ was found to be (surgery type ^(minor)^ * 0.144) + (ISWT ^(% of predicted)^ * 0.948). The OR from robust univariate and multivariable logistic regression for the occurrence of major complications is displayed in Table 3.

**Table 3:** Uni- and multivariate logistic robust regression association between pre- and perioperative variables and complications Clavien–Dindo grade ≥ III

Variable	OR	95% CI	***p***-value
**Gender** (female)	**0.952**	**0.226**–4.006	.947
**Age** (years)	1.017	0.944–1.096	.650
**BMI** (kg/cm^2^)	**0.714**	**0.521–0.979**	.036*
**Living situation** (living together)	0.706	0.129–3.868	.688
**Working status** (works)	1.250	0.507–3.081	.627
**Education level** (high)	1.900	0.759–4.759	.171
**Alcohol consumption** (above norm)	0.533	0.142–1.996	.351
**Smoking status** (no)	0.893	0.273–2.914	.851
**MDASI** total (avg, *n*=28)	0.980	0.728–1.319	.893
**MDASI** symptom (avg, *n*=28)	0.937	0.680–1.290	.688
**MDASI** activity (avg, *n*=28)	1.016	0.827–1.249	.879
**ISWT** (m)	0.998	0.996–1.001	.066
**ISWT** (% of predicted)	**0.981**	**0.967–0.995**	.008*
**Aerobic fitness norm** (labeled fit)	0.875	0.332–2.304	.787
**Time spend sedentary** (min)	0.999	0.997–1.001	.321
**Daily MVPA**—total accumulated (min)	0.996	0.976–1.017	.745
**Daily MVPA**—10-min bouts (min)	0.991	0.949–1.036	.703
**Laparoscopic/closed surgery** (laparoscopic)	0.242	0.034–1.720	.156
**Major/minor surgery** (minor)	**0.240**	**0.060–0.953**	.043*
**Multivariate**			
**Major/minor surgery** (minor)	**0.144**	**0.024–0.858**	.033*
**ISWT** (% of predicted)	0.984	0.954–1.015	.309

*OR* Odds ratio, *95% CI* 95% confidence interval, *BMI* Body mass index, *avg.* Average, *ISWT* Incremental Shuttle Walk Test, *MDASI* MD Anderson Symptom Inventory, *MVPA* Moderate to vigorous physical activity

* *p*≤.05

### Time to functional recovery

The median time to FR was 8 (IQR 5–12) days, ranging from 2 till 56 days. Higher MVPA in both total accumulated bouts (−0.07 less days per minute increase, *p*= .009) and 10-min bouts (−0.14 less days per minute increase, *p*=.007), a minor surgery procedure (−6.39 less days, *p*≤.001), and a higher BMI (−0.46 less days per kg/cm^2^ increase, *p*=.006) resulted in less time to FR. The multivariable model yields an adj. *R*^2^ .43; the model is as follows 12.54 + (MVPA ^(minutes)^ * −.08) + (surgery size ^(1 if minor, 0 if major)^ * −5.64). The association between MVPA in 10-min bouts and time to FR in the subset where major complications occurred was −0.352 less days to FR per minute increase (*R*^2^=.460, *p*=.023). Time to FR analysis is displayed in Table 4.

**Table 4:** Uni- and multivariate robust regression association with time to functional recovery

Variable	Estimate	Std. error	***R***^**2**^	***t***-value	***p***-value	Adj. ***R***^**2**^
**Gender** (female)	0.754	2.206	**0.006**	0.342	.735	
**Age** (years)	0.140	0.097	0.097	1.449	.158	
**BMI** (kg/cm^2^)	**−0.463**	**0.156**	**0.206**	**−2.974**	.006*	
**Living situation** (together)	0.902	1.979	0.005	0.456	.652	
**Working status** (employed)	**−**0.655	1.903	0.004	**−**0.344	.733	
**Education level** (high)	0.365	2.343	0.001	0.156	.877	
**Alcohol norm** (above)	**−**1.255	2.061	0.014	**−**0.609	.547	
**Smoking status** (no)	2.942	2.882	0.044	1.021	.316	
**MDASI** total (avg., *n*=27)	0.269	0.390	0.011	0.691	.496	
**MDASI** symptoms (avg, *n*=27)	0.166	0.399	0.004	0.417	.680	
**MDASI** activities (avg, *n*=27)	0.425	0.379	0.031	1.124	.272	
**ISWT** (m)	**−**0.007	0.004	0.098	**−**1.679	.104	
**ISWT** (% of predicted)	**−**0.054	0.036	0.076	**−**1.508	.142	
**Aerobic fitness norm** (labeled fit)	**−**2.172	1.862	0.044	**−**1.167	.253	
**Time spend sedentary** (min)	**−**0.006	0.004	0.056	**−**1.345	.189	
**Daily MVPA**—total accumulated (min)	**−0.068**	**0.024**	**0.157**	**−2.793**	.009*	
**Daily MVPA**—10-min bouts (min)	**−0.145**	**0.050**	**0.174**	**−2.905**	.007*	
**Laparoscopic/open surgery** (laparoscopic)	**−**1.555	2.045	0.021	**−**0.761	.453	
**Major/minor surgery** (minor)	**−6.392**	**1.638**	**0.426**	**−3.902**	<.001*	
**Multivariate**						
Constant	12.545	2.062		6.083	<.001*	.432
**Major/minor surgery** (minor)	**−**5.643	1.803		**−**3.130	.004*	
**Daily MVPA**—10-min bouts (min)	**−**0.079	0.031		**−**2.573	.016*	
**Major complications subset analysis**						
**Daily MVPA** – 10-minute bouts (min)	**−0.352**	**0.126**	**0.460**	**−2.798**	.023*	

*BMI* body mass index, *ISWT* Incremental Shuttle Walk Test, *MDASI* MD Anderson Symptom Inventory, *MVPA* moderate to vigorous physical activity

* *p*≤.05

## Discussion

To our knowledge, this is the first study investigating device-measured MVPA levels in HPB resection candidates not receiving PA interventions. Patients scheduled for HPB surgery engage in low daily MVPA at baseline while waiting for surgery. Furthermore, a relation was found between the level of MVPA and time to FR after HPB surgery for (pre)malignancy; patients with higher levels of PA require less time to FR. The current findings suggest that increasing a patient’s preoperative MVPA level might be an intervention to improve the postsurgical outcome.

### Physical activity

The median MVPA level measured in the current study was low but comparable to other preoperative activity monitor measured MVPA studies, e.g., gastric bypass and lumbar fusion surgery (Van der Meij et al. 2017; Lotzke et al. 2018). However, this comparison is somewhat arbitrary due to the influence of age and the variety in symptom burden experienced among different pathologies. Furthermore, the variety in activity monitor device configuration like MVPA cutoff point and wear-time validation highly influences the results (Gorman et al. 2014). Nevertheless, this study demonstrates that the majority (79%) of the patients enlisted for HPB surgery did not perform sufficient MVPA to meet the guideline of 150 min MVPA per week (Weggemans et al. 2018; WHO 2009).

These findings might be explained by the psychological impact of being enlisted for surgery because of malignancy. Namely, being informed about the presence of a tumor can result in changes in PA behavior (Allender et al. 2008). Participants were recruited directly after being enlisted, and measurements were performed during the first week after enlistment. Due to the design of the study, it remains unclear whether the measured level of MVPA and performed distance covered on the ISWT is temporary. These values might reach higher levels once the impact of the news diminishes. Previous studies have reported an increase in PA during the waiting period (Kim et al. 2009). The observed increase might have been caused by an increased awareness or social desirability of the participant, as they had to fill out PA questionnaires, wear a PA monitor, or perform physical fitness measures during this study. Furthermore, it seems likely that patients perform less MVPA due to the interference of tumor-related symptoms. However, there was no evidence for an association between the experienced symptom burden like pain and fatigue, measured with the MDASI, and the level of performed MVPA. Notably, participants experienced fairly low symptom interference in our study, 1.87 points on mean out of 10. It therefore seems probable that subjects with high symptom interference were more likely to reject study participation. Due to the small sample size, no subcategory analysis with subjects experiencing high levels of symptom burden could be performed.

### Postoperative outcomes

A significant association was found between MVPA and time to FR (*R*^2^= 0.17, *p*= .006) but no significant association was found between MVPA and the occurrence of postoperative complications (OR = 0.99, 95% CI= 0.95–1.03, *p*= .67). These findings are in accordance with the systematic review and meta-analysis in preoperative cancer patients by Steffens et al., who found an association between higher levels of preoperative MVPA and a shorter absolute LOS (OR=3.66; 95% CI= 1.38 to 9.6), but not with postoperative complications (OR=2.60; 95% CI=0.59 to 11.37). The majority of studies in this meta-analysis used self-reported MVPA and participants undergoing neo-adjuvant (physical) therapy (Steffens et al. 2019). However, the meta-analysis as well as the current study consistently indicates that higher levels of MVPA positively influence a patient’s capacity to endure the demands of surgery (Steffens et al. 2019).

A subject’s level of preoperative MVPA was associated with reduced time to FR; 43% of the time variance to FR could be explained via multivariable robust regression including surgery size and MVPA levels. This reduction might be explained by the lower relative capacity needed to perform activities in daily living by patients with higher levels of aerobic fitness. FR is determined by both functional and physiological criteria, that is, higher levels of aerobic fitness increase a patient’s functional capacity to perform activities of daily living (Jackson et al. 2009). However, caution is needed when interpreting these results since we did not directly measure aerobic reserves at the moment of FR.

Furthermore, a higher percentage of predicted distance covered on the ISWT was associated with reduced OR for the occurrence of major postoperative complications found by univariate robust regression. Similar reductions have been reported in multiple studies among a large variety of surgical procedures (Kumar and Garcea 2018; Snowden et al. 2010; Simões et al. 2018; Van Beijsterveld et al. 2019; Levett and Grocott 2015). These reductions might be explained by the higher aerobic reserves enhancing the body’s capacity to cope with the responses to the surgical procedure. Nevertheless, a higher percentage of predicted distance covered on the ISWT was not found to have a significant association in multivariable robust regression including surgery size. Notably, the current study found lower OR for the occurrence of major complications in subjects with a higher BMI. This result is inconsistent to previous studies showing increased OR for the development of major complications in obese and overweight subjects undergoing pancreatectomy procedures (Lovasik et al. 2019). This difference might be explained by the overrepresentation of subjects with high BMI scores undergoing a major surgery procedure in the present study (Wilcoxon rank sum test, *W* = 74, *p*= .02). Major surgery has a higher risk of resulting in major complications. Therefore, BMI was removed from multivariable regression analysis in the current study. Additionally, we found a reduction in time to FR after major complications in subjects performing higher levels of PA. Therefore, it could be concluded that because subjects with a higher level of MVPA have more capacity to cope with the demands endured by complications, the impact of complications is less. Nevertheless, these results should be interpreted with some caution since only nine subjects reached FR after major complications.

### Treatment opportunity

This study identifies preoperative MVPA as a modifiable patient factor to reduce time to FR. Multiple associations between performed MVPA and preoperative variables were found, namely, MVPA decreased with advancing age with 0.52 min per age year (*p*≤.001) and increased in participants with higher aerobic fitness, covering more distance during the ISWT (0.02 min per m, *p*=.008). Since both PA and aerobic fitness decline with age (Milanović et al. 2013), these findings underpin the hypothesis that unfit and older patients could benefit most from interventions aimed to improve aerobic fitness and to increase MVPA levels, especially in the waiting time before surgery. Furthermore, although this study does not include a detailed cost analysis, increasing the level of preoperative MVPA via relative low-cost treatment modalities as education, wearables, and physiotherapy may be of particular relevance for the reduction of hospital costs due to the shorter hospital stay (see supplementary data) (Straatman et al. 2015).

### Limitations

There are some limitations to this observational pilot study. The first is that no pre-trail sample-size calculation was performed. This can impact the results with a higher risk of type II errors. Since a limited number of HPB resections are yearly performed, participants were included via convenience sampling in a multi-center design. However, the final sample size obtained is comparable with several other studies aimed at measuring PA via activity monitor devices in major abdominal surgery (Mungovan et al. 2013; Dronkers et al. 2013). Unfortunately, only 26% of the approached subjects provided consent to participate in the study. A reason for this low participation rate might have been the moment of inclusion, namely, directly after being enlisted for surgery. Frequently mentioned reasons for declining participation were the feeling of being emotionally overwhelmed and currently not having the energy to endorse participation. These reasons might have induced a sample slightly biased in the direction of somewhat fitter patients. Larger sample sizes and less strenuous PA measurements can be more easily acquired via questionnaires. Nevertheless, activity monitor measured PA is a feasible and more reliable method of determining PA and is therefore recommended (Steffens et al. 2019; Van der Meij et al. 2017).

## Conclusion

This study demonstrates that 79% of the patients enlisted for resection of HPB (pre) malignancy performed insufficient MVPA. A higher level of MVPA, objectively measured with an activity monitor, was independently associated with a shorter time to FR. However, levels of MVPA were not associated with postoperative complications. Stimulating MVPA in the waiting time for surgery might help to reduce the LOS. These findings add to a growing body of evidence suggesting that higher levels of MVPA positively influence a patient’s capacity to endure the demands of surgery and improve the outcome of surgery.

## Supplementary Information

**Additional file 1: Supplementary table.** Uni- and multivariate robust regression association with absolute length of hospital stay.

## Data Availability

The datasets used and analyzed during the current study are available from the corresponding author on reasonable request.

## Abbreviations

MVPA: Moderate to vigorous physical activity
PA: Physical activity
HPB: Hepato-pancreato-biliary
LOS: Length of hospital stay
FR: Functional recovery
ISWT: Incremental Shuttle Walk Test
MDASI: MD Anderson Symptom Inventory
BMI: Body mass index
OR: Odds ratio
IQR: Interquartile range
