# Supplementary material for: Objectively measured preoperative physical activity is associated with time to functional recovery after hepato-pancreato-biliary cancer surgery: a pilot study
Source: Perioper Med (Lond). 2021 Oct 4;10:33. doi: 10.1186/s13741-021-00202-7 (PMC8489102; doi:10.1186/s13741-021-00202-7)
Supplement: Supplementary file 1 — Additional file 1: Supplementary table. Uni- and multivariate robust regression association with absolute length of hospital stay. [file 13741_2021_202_MOESM1_ESM.pdf]

Supplementary table

Uni- and multivariate robust regression association with absolute length of hospital stay

Variable		Estimate	Std. error	R^2	t-value	p-value	Adj. R^2
**Gender**	(female)	-0.464	2.146	0.002	-0.216	.830	
**Age**	(years)	0.146	0.114	0.070	1.288	.208	
**BMI**	(kg/cm^2)	-0.394	0.214	0.120	-1.845	.075	
**Living situation**	(together)	1.522	2.056	0.013	0.740	.465	
**Working status**	(employed)	-1.158	2.087	0.011	-0.555	.583	
**Education level**	(high)	0.692	2.612	0.004	0.265	.793	
**Alcohol Norm**	(above)	-1.473	2.273	0.017	-0.648	.522	
**Smoking status**	(no)	2.313	3.275	0.022	0.706	.486	
**ISWT**	(meters)	-0.007	0.004	0.100	-1.709	.098	
**ISWT**	(% of predicted)	**-0.076**	**0.035**	**0.132**	**-2.177**	**.038***	
**MDASI** total	(avg., n=27)	-0.047	0.455	0.000	-0.104	.918	
**MDASI** symptoms	(avg, n=27)	-0.174	0.449	0.004	-0.388	.701	
**MDASI** activities	(avg, n=27)	0.118	0.368	0.025	0.321	.751	
**Time spend sedentary**	(minutes)	-0.008	0.005	0.089	-1.760	.089	
**Daily MVPA** - total accumulated	(minutes)	**-0.070**	**0.027**	**0.132**	**-2.561**	**.016***	
**Daily MVPA** - 10-minute bouts	(minutes)	**-0.150**	**0.058**	**0.149**	**-2.574**	**.015***	
**Laparoscopic / open surgery**	(Laparoscopic)	-2.426	2.086	0.043	-1.163	.254	
**Major / minor surgery**	(minor)	**-7.063**	**1.688**	**0.489**	**-4.185**	**<.001***	
**Multivariate**							
Constant		17.184	2.359		7.285	<.001*	.533
**ISWT**	(% of predicted)	-0.051	0.024		-2.095	.045*	
**Major / minor surgery**	(minor)	-6.609	1.349		-4.897	<.001*	
**Daily MVPA** - 10-minute bouts	(minutes)	-0.035	0.031		-1.147	.261	

BMI = Body Mass Index, ISWT = Incremental Shuttle Walk Test, MDASI = MD Anderson Symptom Inventory, MVPA = Moderate to Vigorous Physical Activity, * = P ≤.05, avg. = average
